# Endometrial aging and uterine receptivity: endometrial receptivity analysis (ERA) outcomes in female patients of diverse age groups

**DOI:** 10.1007/s10815-026-03824-2

**Published:** 2026-02-09

**Authors:** Kaia M. Schwartz, Bahar D. Yilmaz, Meagan Chan, Marcelle I. Cedars, Hakan Cakmak, David Huang

**Affiliations:** Division of Reproductive Endocrinology and Infertility, Department of Ob/Gyn and Reproductive Sciences, University of California, San Francisco, 499 Illinois Street, 6th Floor, San Francisco, CA 94158 USA

**Keywords:** Endometrium, Receptivity, Aging, Implantation

## Abstract

**Purpose:**

To assess whether the endometrial receptivity analysis (ERA) captures receptivity changes attributed to endometrial aging and whether it may be useful for older patients undergoing fertility treatment.

**Methods:**

Retrospective cohort study of patients who underwent ERA testing at an academic center (01/2019–05/2024). The ERA inferred transcriptomic levels of canonical receptivity markers from biopsies obtained at standard timing. Demographic and treatment-related variables were analyzed by age group. The proportion of non-receptive ERA results (pre- and post-receptive combined) was compared using Fisher’s exact test. Univariable and multivariable logistic regression assessed associations between predictors and non-receptive ERA.

**Results:**

Of 210 patients, 205 were included. Age distribution was < 35 (*n* = 35, 17%), 35–37 (*n* = 58, 28.3%), 38–40 (*n* = 53, 25.8%), and ≥ 41 (*n* = 59, 28.8%). Overall, 166 (81.0%) ERAs were receptive, 33 (16.1%) pre-receptive, and 6 (2.9%) post-receptive. BMI, infertility diagnosis, and prior implantation or miscarriage history did not differ by age. Non-receptive ERA proportions were 20% (< 35), 17.2% (35–37), 17.0% (38–40), and 22.0% (≥ 41) (*p* = 0.52). In multivariable analysis adjusting for BMI and number of prior failed euploid transfers, age was not associated with non-receptive ERA (aOR 0.98, 95% CI 0.34–2.30, *p* = 0.97).

**Conclusion:**

Uterine age was not associated with increased odds of non-receptive ERA, suggesting that the test does not capture age-related changes in endometrial receptivity. Although endometrial aging is implicated in reduced embryo transfer success, the ERA should not be ordered solely on the basis of uterine age. The ERA may not reliably detect age-related endometrial differences in the window of implantation at a clinical level.

**Supplementary Information:**

The online version contains supplementary material available at 10.1007/s10815-026-03824-2.

## Introduction

Women are increasingly delaying childbearing worldwide, especially in developed nations. In the USA, the average maternal age rose from 24.6 in 1970 to 27.2 in 2000, with an even greater increase in urban areas [1]. By 2017, the average maternal age at first birth in large urban counties was 29.0 [2]. This trend is driven by factors such as career and educational priorities, greater contraceptive access, relatively limited awareness of age-related fertility decline, and personal choice [3].

While the exact age at which fertility begins to rapidly decline is somewhat variable, it is well established that after age 35, ovarian reserve and oocyte quality diminish, leading to increased chromosome missegregation, lower pregnancy rates, and higher miscarriage rates [4–6]. Preimplantation genetic testing for aneuploidy (PGT-A) and oocyte donation have become more widely used to mitigate these effects of ovarian aging. However, live birth rates following in vitro fertilization treatment have now plateaued around 50–60% per euploid embryo transfer, with emerging research suggesting that endometrial aging may also contribute to implantation failure, miscarriage, and lower clinical pregnancy rates in women over 35 [7–9].

Traditionally, the uterus and endometrium were thought to age more slowly—or perhaps, less clinically significantly—than the ovaries. The effects of endometrial aging have been comparatively overlooked in the literature compared to ovarian aging. However, recent studies challenge this view, showing age-related epigenetic alterations in the endometrium [10, 11]. In animal models, genome-wide analysis has revealed declining estrogen and progesterone receptor expression with age, potentially disrupting normal endometrial function [12, 13]. This, in turn, could lead to perturbations in biological pathways that critically depend on steroid hormone action, such as endometrial decidualization and the establishment of the window of implantation (WOI). Human clinical studies further support this notion, demonstrating a potentially inverse relationship between maternal age and endometrial function. A prospective study in oocyte donor recipients found that pregnancy rates declined and miscarriage rates increased with recipient age, implicating uterine aging in implantation failure [14]. Similarly, another study that examined pregnancy outcomes of recipients sharing the same pool of oocytes from young donors observed that younger recipients exhibited better outcomes compared to recipients over the age of 40 [15]. Interestingly, basic science studies have also identified potential molecular signatures associated with endometrial senescence in women under age 35 following recurrent implantation failure, highlighting endometrial biological aging independent of chronological age as a possible contributor to implantation failure [16].

The WOI opens in the early to mid-luteal phase, when the endometrium is considered optimally receptive for embryo implantation. There has been a focus on classifying the gene expression pattern within the endometrium during this critical period to define a clinically relevant signature for the WOI [17–20]. Some have proposed that dyssynchrony between the embryo and the WOI endometrium can result in implantation failure or miscarriage if an embryo is transferred too early (pre-receptive) or too late (post-receptive) [21, 22]. The endometrial receptivity analysis (ERA) assesses transcriptomic profiles of 238 genes identified from healthy controls to help clinicians gauge whether the endometrium displays a molecular signature that is pre-receptive, receptive, or post-receptive in relation to the WOI signature [20]. This clinical tool purports to provide personalized embryo transfer timing based on the patient’s specific receptivity profile in relation to the duration of progesterone exposure [23]. Of note, data regarding the practical applications of the ERA test remain mixed, and the appropriate indication and patient population that could benefit from the ERA remain to be determined [24–26]. Although the clinical utility of the ERA for the general infertility patient population appears limited [26–32], a recent study showed transcriptomic evidence of suboptimal endometrial receptivity as well as changes in endometrial cellular composition with advanced maternal age [33]. Additionally, reports have demonstrated an association between advancing maternal age and reduced implantation rates, even in the context of frozen embryo transfers with exclusively euploid embryos or embryos derived from donor oocytes [34, 35]. These data raise renewed focus on the endometrium impacting implantation and may focus consideration of the ERA as a tool to evaluate implantation timing based on maternal age, underscoring the importance of further investigation in this area.

Given the previously reported molecular, cellular, and epigenetic changes observed in the endometrium of women with advanced reproductive age, we hypothesize that there could be a higher prevalence of WOI displacement with increasing female age. If this hypothesis is confirmed, there may be utility in employing the ERA specifically in older female patients to guide embryo transfer timing and aid reproductive outcomes. Herein, our study aims to investigate whether increasing female chronological age is associated with a higher prevalence of non-receptive ERA secondary to endometrial aging.

## Methods

### Population

This retrospective cohort study included patients seeking fertility treatment at a single academic institution, the University of California, San Francisco (UCSF). Between 01/2019 and 05/2024, 210 patients underwent endometrial biopsy for the endometrial receptivity analysis (ERA). Five patients were excluded from the analysis: three due to insufficient biopsy material, one due to a “non-informative” result, and one due to a compromised sample during transit. Demographic information, including age, body mass index (BMI), infertility diagnosis, and pregnancy history at the time of biopsy, was obtained through chart review. Additionally, data on the number of prior implantation failures, including the total number of embryos transferred and the total number of euploid embryos transferred, were recorded for each patient. Implantation failure was defined as a negative serum human chorionic gonadotropin (hCG) following embryo transfer. This study was approved by the UCSF Institutional Review Board.

### Endometrial biopsy

Standard technique for endometrial biopsy was performed using a Pipelle catheter (CooperSurgical, Trumbull, CT). The biopsies were performed in the luteal phase under three different endometrial preparation methods: natural cycle (NC), modified natural cycle (mNC), and programmed cycle (PC). The timing of the biopsy was performed according to the instructions provided by the manufacturer of the ERA test.

At our center, ERA testing is primarily performed under programmed cycle conditions. However, there was a minority of patients who underwent ERA biopsy in either a natural cycle (NC) (*n* = 10; 4.8%) or a modified natural cycle (mNC) (*n* = 25; 12.2%). The programmed cycle group received exogenous estradiol either in the form of estradiol patches (300 mcg every 3 days) or oral Estrace 6 mg daily to stimulate endometrial lining development. There was a minimum of 12 days of estrogen-only phase before the patients were brought in for a transvaginal ultrasound to evaluate the endometrial lining. After an ultrasound evaluation of the endometrium, progesterone (IM Progesterone in oil, 50 mg daily) was initiated. Endometrial biopsy in the PC group was performed on the 6th day of progesterone therapy.

In both NC and mNC endometrial preparation groups, the endometrial biopsy was performed either at luteinizing hormone (LH) surge + 6 days or 7 days after administration of choriogonadotropin alfa, respectively (EMD Serono, Rockland, MA). The mNC group also received luteal phase vaginal progesterone support (Prometrium 200 mg twice daily or Endometrin 100 mg twice daily). All endometrial biopsy samples were processed and sent to Igenomix according to the manufacturer’s instructions for ERA testing.

### Statistical analysis

The primary outcome of this study was the proportion of non-receptive ERA results (pre- and post-receptive combined) stratified by female age group. Demographic, treatment parameters, and embryo transfer outcomes were compared across age groups using the Kruskal–Wallis test, chi-squared test, or Fisher’s exact test as appropriate. The proportions of non-receptive ERA results by age group were compared using Fisher’s exact test. A sensitivity analysis, including only patients who were biopsied for ERA during a programmed cycle, was also performed. Univariable logistic regression analyses were performed to identify potential predictors of a non-receptive ERA result. Baseline- and treatment-related characteristics that were significantly different among age groups, and factor(s) with a significance level of *p* < 0.08 in the univariable analyses, were included in a multivariable logistic regression model. An additional multivariable logistic regression was performed that included BMI due to biologic plausibility that this variable could affect our outcome of interest. All statistical tests were two-sided, and significance was defined as p < 0.05. Analyses were conducted using Stata v18.0 (StataCorp, College Station, TX).

## Results

A total of 210 patients underwent endometrial sampling for ERA testing during the study period. Five patients were excluded from the analysis due to inconclusive testing. Of the remaining 205 ERA results, 166 (81.0%) were classified as “receptive,” while 39 (19.0%) were non-receptive (pre- or post-receptive). Among the non-receptive results, 6 (2.9%) were “post-receptive” and 33 (16.1%) were “pre-receptive.” Demographic and treatment parameters by age group category are shown in Table 1. The median age in the youngest group (age < 35) was 33 (interquartile range; IQR 32–34); in the 35–37 group, median (IQR) age was 36 (35–37); in the 38–40 group, median (IQR) age was 39 (38–40); and in the oldest group (age ≥ 41), the median age (IQR) was 43 (42–44) (*p* < 0.01). The median BMI was not significantly different among the age groups: in the youngest group, median BMI was 23.1 (IQR 21.8–25.5), age 35–37 median BMI was 22.5 (IQR 21.3–26.5), age 38–40 median BMI was 23.2 (IQR 21.8–26.6), and the median BMI in the age ≥ 41 group was 24.7 (IQR 22.0–27.5) (*p* = 0.20). The distributions of nulliparity status, number of previous miscarriages, and primary infertility diagnosis were not statistically different across age groups (all *p* > 0.05). Additionally, the number of previous implantation failures was similar across all age groups: in the < 35 age group, median was 1 (IQR 1–2), age 35–37 median was 2 (IQR 1–2), age 38–40 median was 1 (IQR 1–2), and for the ≥ 41 age group, the median was 1 (IQR 0–2) (*p* = 0.75). The number of previous failed euploid embryo(s) transferred approached but did not meet statistical significance. The youngest group (age < 35) had a median of 0 previous failed euploid embryo transfers (IQR 0–2). For the next age group (35–37), the median was 1 (IQR 0–1); similarly, for the group aged 38–40, median was 1 (IQR 0–2); and for the oldest group (age 41 and above), the median was 0 (IQR 0–1) (*p* = 0.054).

**Table 1:** Patient characteristics by age category

Demographics	< 35 (*N* = 35)	35–37 (*N* = 58)	38–40 (*N* = 53)	41 and above (*N* = 59)	*p*-value
**Age**^**a**^	33 (32, 34)	36 (35, 37)	39 (38, 40)	43 (42, 44)	** < 0.01**
**BMI**^**a**^	23.1 (21.8, 25.5)	22.5 (21.3, 26.5)	23.2 (21.8, 26.6)	24.7 (22.0, 27.5)	0.20
**Nulliparous**^**b**^					
Yes	30 (85.7%)	52 (89.7%)	40 (75.5%)	46 (78.0%)	0.19
No	5 (14.3%)	6 (10.3%)	13 (24.5%)	13 (22.0%)	
**Number of previous miscarriages**^**a**^	0 (0, 1)	0 (0, 2)	0 (0, 1)	1 (0, 1)	0.20
**Primary infertility diagnosis**^**b**^					
Unexplained	20 (57.1%)	37 (63.8%)	29 (54.7%)	37 (63.8%)	0.64
Polycystic ovary syndrome	3 (8.6%)	2 (3.5%)	3 (5.7%)	3 (5.2%)	
Male factor	3 (8.6%)	5 (8.6%)	6 (11.3%)	8 (13.8%)	
Tubal factor	1 (2.9%)	3 (5.2%)	1 (1.9%)	1 (1.7%)	
Endometriosis	0 (0%)	2 (3.5%)	0 (0%)	0 (0%)	
Uterine	1 (2.9%)	3 (5.2%)	2 (3.8%)	1 (1.7%)	
Recurrent pregnancy loss	1 (2.9%)	3 (5.2%)	1 (1.9%)	4 (6.8%)	
Other*	6 (17.1%)	3 (5.2%)	11 (20.8%)	5 (8.6%)	
**Number of implantation failures**^**a**^	1 (1, 2)	2 (1, 2)	1 (1,2)	1 (0, 2)	0.75
**No. of failed euploid embryos transferred**^**a**^	0 (0, 2)	1 (0, 1)	1 (0, 2)	0 (0, 1)	0.054

^a^Data presented as median (25th, 75th percentile)

^b^Data presented as *n* (%)

^*^Other includes diminished ovarian reserve, advanced reproductive age of female, primary ovarian insufficiency, history of gonadotoxic chemotherapy, need for donor gamete(s), and inability to have intercourse

### The proportion of non-receptive ERA by age category

Endometrial receptivity analysis (ERA) results by age category are shown in Table 2. In the age < 35 group (*n* = 35), the majority of ERA results were receptive (*n* = 28; 80%). Of the non-receptive results in this age group (*n* = 7; 20.0%), 6 were pre-receptive (17.1%) and one was post-receptive (2.9%). In the age 35–37 group (*n* = 58), 48 biopsies were receptive (82.8%). In this age group, 10 biopsies (17.2%) were non-receptive: 7 were pre-receptive (12.0%) and 3 were post-receptive (5.2%). The age 38–40 group (*n* = 53) had 44 receptive (83.0%) and 9 non-receptive ERA (17.0%) results, of which 7 were pre-receptive (13.2%) and 2 were post-receptive (3.8%). The age 41 and above group (*n* = 59) consisted of 46 receptive results (78.0%) and 13 non-receptive (22.0%) results, all of which were pre-receptive. There was no significant difference in the proportions of non-receptive ERA results by age group (*p* = 0.52). Additionally, a sensitivity analysis was performed, including only patients who were biopsied for ERA during a programmed cycle. There was still no significant difference in the proportion of non-receptive ERA results by age group (*p* = 0.25). These results can be found in Supplemental Table 3.

**Table 2:** Proportion of non-receptive ERA by age category

	< 35 (N = 35)	35–37 (*N* = 58)	38–40 (*N* = 53)	41 and above (*N* = 59)	*p*-value
**Non-receptive ERA**	7 (20.0%)	10 (17.2%)	9 (17.0%)	13 (22.0%)	0.52
Pre-receptive	6 (17.1%)	7 (12.0%)	7 (13.2%)	13 (22.0%)	
Post-receptive	1 (2.9%)	3 (5.2%)	2 (3.8%)	0 (0%)	
**Receptive ERA**	28 (80%)	48 (82.8%)	44 (83.0%)	46 (78.0%)	

### Logistic regression to assess associations between age group and non-receptive ERA

Univariable logistic regression was first performed to evaluate potential associations between individual patient demographics or treatment-related factors and a non-receptive ERA result. As shown in Table 3, no significant associations were found between older age groups and non-receptive ERA. Compared to the reference group (age < 35), those aged 35–37 had a non-significant increased odds of a non-receptive ERA (odds ratio; OR 1.20 [95% CI 0.41–3.51], *p* = 0.74). Similarly, age 38–40 had non-significant, mildly increased odds of a non-receptive ERA (OR 1.22 [95% CI 0.41–3.66], *p* = 0.72). The oldest group, age 41 and above, had a nonsignificant decrease in odds of a non-receptive ERA (OR 0.88 [95% CI 0.32–2.48], *p* = 0.82). BMI, primary infertility diagnosis, number of previous miscarriages, number of implantation failures, and number of failed euploid or total embryos transferred preceding the endometrial biopsy were not significantly associated with higher odds of non-receptive ERA in our cohort (all *p* > 0.05). Given the trend toward a difference in the number of prior failed euploid embryo transfers across age groups (*p* = 0.054) and its potential association with ERA outcomes, this variable was included in a multivariable logistic regression model. After adjusting for this factor, there remained no statistically significant difference in non-receptive ERA results by age category. Compared to the reference group (age < 35), the adjusted OR (95% CI) for a non-receptive ERA was aOR 1.21, (95% CI 0.41–3.55) for age 35–37, aOR 1.23 (95% CI 0.41–3.69) for age 38–40, and aOR 0.84 (95% CI 0.30–2.38) for age 41 and above (all *p* > 0.05). An additional multivariable logistic regression model was conducted, including both the number of prior failed euploid embryo transfers and BMI, as there is biologic plausibility that BMI could affect the hormonal milieu of the uterus. There remained no significant difference in the outcome of non-receptive ERA. Compared to the reference group (age < 35), the aOR (95% CI) for a non-receptive ERA was 1.23 (0.42–3.6) for age 35–37, aOR 1.30 (95% CI 0.43–4.0) for age 38–40, and aOR 0.98 (95% CI 0.34–2.8) for age 41 and above (all *p* > 0.05).

**Table 3:** Logistic regression analyses of possible predictors of non-receptive ERA

Variable	Unadjusted OR—univariable logistic regression		Adjusted*OR—multivariable logistic regression		Adjusted** OR—multivariable logistic regression	
	OR (95% CI)	*p*-value	OR (95% CI)	*p*-value	OR (95% CI)	*p*-value
**Age category (compared to age < 35)**						
Age 35–37	1.20 (0.41–3.51)	0.74	1.21 (0.41–3.55)	0.73	1.23 (0.42–3.63)	0.70
Age 38–40	1.22 (0.41–3.66)	0.72	1.23 (0.41–3.69)	0.71	1.30 (0.43–3.93)	0.64
Age 41 and over	0.88 (0.32–2.48)	0.82	0.84 (0.30–2.38)	0.75	0.98 (0.34–2.85)	0.97
**BMI**	0.96 (0.90–1.03)	0.24			0.96 (0.90–1.03)	0.22
**Primary infertility diagnosis**	0.97 (0.86–1.09)	0.57				
**No. previous miscarriages**	1.06 (0.75–1.48)	0.75				
**No. implantation failures**	1.07 (0.79–1.44)	0.67				
**No. failed euploid embryo(s) transferred**	0.87 (0.61–1.23)	0.43	0.85 (0.60–1.21)	0.36		
**No. total failed embryo(s) transferred**	0.98 (0.81–1.20)	0.86				

^*^Adjusted for the number of failed euploid embryo transfers

^**^Adjusted for the number of failed euploid embryo transfers and BMI

Pregnancy outcomes in those who completed a subsequent FET after ERA testing are shown in Supplemental Table 1. There were no significant differences in implantation, clinical pregnancy, or live birth rates in the subsequent FET cycle in women < 35 as compared to older age groups. When the data was limited to only patients who had a non-receptive ERA result, there was again no difference in pregnancy outcomes between the reference group (age < 35) and older age groups with ERA-guided timing (Supplemental Table 2).

## Discussion

The field of assisted reproductive technology has made strides in addressing infertility associated with ovarian aging using in vitro fertilization, pre-implantation genetic testing for aneuploidy, and oocyte donation. However, critical determinants of success from the uterine perspective and the endometrium—the soil for implantation—have remained largely elusive and controversial despite significant efforts. In particular, there is a growing focus on endometrial aging as a potential target for improving pregnancy rates and healthy live births, especially as the age of motherhood in modern society continues to rise [36]. Given the potential impact of chronological aging on the cellular machinery associated with endometrial remodeling, we investigated whether advanced female reproductive age is associated with aberrant expression of canonical receptivity markers on the ERA, many of which relate to decidualization and progesterone effects. Contrary to what we hypothesized, our study did not find increased odds of non-receptive ERA results associated with increased female age.

Animal studies have demonstrated an inverse relationship between age and estrogen and progesterone receptor density in the endometrium, suggesting that endometrial tissue may become less hormone-sensitive with increasing age [12, 13]. This observation provides biologic plausibility for impaired uterine receptivity with aging, as implantation depends on hormone-mediated endometrial remodeling. For example, thinner endometrial lining has been observed in older females [37], and mean serum estradiol levels also decline with advancing age [38, 39]. Several observational studies also reported declining pregnancy and live birth rates in oocyte recipients as recipient age increases [34, 40, 41], although there is no consensus on the threshold age in humans at which uterine receptivity steadily declines [30–32].

The endometrial receptivity analysis (ERA) test was developed to assess the transcriptomic profile of 238 genes that largely represent canonical receptivity markers that were extrapolated from menstrual cycle phase and logically reflect downstream effects of progesterone. Importantly, recent high-quality data raise substantial concerns about the lack of benefit with ERA. A large randomized controlled trial with single euploid frozen embryo transfers (FETs) demonstrated no significant improvement in pregnancy or live birth rates when embryo transfers occurred at the timing recommended by the ERA compared to standard timing [42]. Similarly, a meta-analysis by Zolfaroli et al. concluded that ERA-guided transfer does not confer significant advantages [43]. These findings suggest that, while ERA may provide biological insights at the transcriptomic level, its clinical value in improving embryo transfer outcomes appears limited based on current evidence.

Of note, there have been multiple recent publications implicating endometrial aging as a factor affecting implantation success and receptivity; hence, it is worthwhile to assess the utility of the ERA in this specific context [34, 40, 41, 44]. Our study conclusions suggest that the ERA also does not adequately capture potential age-related differences in endometrial receptivity. One possible explanation is that mRNA patterns of a gene panel may not be sensitive enough to detect or report subtle age-related changes in specific genes. It is also possible that the age-related impact occurs at the post-transcriptional stages and instead affects protein levels that may better reflect the functional state of the endometrium. It is also worth noting that the distribution of ERA results in our cohort differed from that reported in several other studies [45, 46]. For example, Barrentexea et al. and others reported an approximately 50–60% “receptive” rate, whereas in our cohort, nearly 80% of ERA tests were “receptive” [27, 32, 46]. Several factors may account for this discrepancy. First, a large majority of our patients (83%) underwent programmed cycles with intramuscular progesterone in oil (50 mg daily), whereas the studies cited above relied exclusively on vaginal progesterone or mixed vaginal/intramuscular combination. Exogenous replacement of steroid hormones may compensate for a reduction in estradiol/progesterone production and/or receptor concentrations or responsiveness with aging. Additionally, serum and endometrial progesterone concentrations differ substantially between vaginal and intramuscular administration routes, potentially altering luteal phase molecular expression and physiology [27, 47, 48]. All ERA samples were processed and analyzed by Igenomix in a blinded fashion such that our institution had no influence on assay execution or result classification. Our findings do not support the notion that older age groups would exhibit a significantly increased proportion of non-receptive ERA results. Advanced reproductive age itself should therefore not be an indication to undergo ERA testing.

Our study was strengthened by its large sample size, diverse age distribution, and relatively uniform treatment protocols within a single center. We did not exclude patients based on infertility diagnosis, which helps augment the generalizability of our results. The ERA test and determination of “endometrial receptivity” were analyzed and reported by a third-party company (Igenomix) that was blinded to the details of a patient’s medical history. Potential confounders for ERA non-receptivity were assessed and adjusted for as appropriate in our analysis.

We also acknowledge the limitations of our study. As a retrospective cohort study, we attempted to reduce bias by making a detailed assessment of demographic and treatment-related parameters that could affect our outcome of interest. Despite our methodology, it is possible that we omitted a variable that exerts an effect on ERA results, given the limited knowledge on proven clinical factors that impact endometrial receptivity on a transcriptomic level. Despite our relatively large cohort size, it is still possible that the sample size did not provide enough power to detect small but significant differences. However, as above, the translational value of the ERA is also being contested [42, 49]. It is possible that clinically relevant endometrial aging is not detectable using this tool. In Supplemental Tables 1 and 2, we evaluated clinical outcomes of the subsequent FET following ERA. Across age groups, and acknowledging the limitations of our sample size, we observed no differences in implantation, clinical pregnancy, or live birth rates, regardless of whether transfer timing was adjusted based on ERA results. Given the relatively small number of pregnancies included, larger cohort studies are needed to validate these findings. Additionally, because our clinical protocol involves adjusting transfer timing when the ERA recommends a change, we do not have comparable data for patients who underwent transfers at standard timing despite a non-receptive result.

Our results highlight the need for additional scientific investigations into the cellular and molecular changes that occur in the aging endometrium, which may be better identified in extreme ranges of reproductive age [28]. Prospective clinical data on IVF outcomes, adjusting for female age, embryo quality, endometrial factors, and treatment history/indication, can further substantiate the phenomenon of endometrial aging. Modern techniques utilizing single-cell sequencing may also provide a more granular view of endometrial changes with chronological aging, particularly in cell types beyond the epithelial and stromal populations [28, 39]. These efforts will help further understand which molecular changes associated with endometrial aging are clinically relevant, and inform therapeutic development on how to best overcome these alterations in the endometrial environment.

## Conclusion

There is biologic plausibility that the WOI environment may change with age. However, the ERA test does not appear to capture this difference. The proportion of non-receptive ERA was not significantly higher in older reproductive age groups, even in those over the age of 41. Alternatively, our results may also support that the WOI is not significantly impacted by age when assessed by transcriptomic levels of canonical receptivity markers. Based on our results, we suggest caution in using the ERA solely on the basis of increased maternal age, as our findings indicate that the ERA may not reliably detect age-related differences in the window of implantation. Rigorous, prospective clinical and basic science studies are needed to further understand endometrial aging and its potential clinical impact in fertility treatment.

## Supplementary Information

Below is the link to the electronic supplementary material.

Supplementary file1 (DOCX 16.9 KB)

## Data Availability

The data is not publicly available. However, we can provide the dataset to the reviewers if necessary.

## References

[1] Mathews TJ, Hamilton BE. Mean age of mother, 1970–2000. 12564162

[2] Products - Data Briefs - Number 320 - September 2018 [Internet]. [cited 2025 Mar 6]. https://www.cdc.gov/nchs/products/databriefs/db323.htm. Accessed 6 Mar 2025

[3] Safdari-Dehcheshmeh F, Noroozi M, Taleghani F, Memar S. Factors influencing the delay in childbearing: a narrative review. Iran J Nurs Midwifery Res [Internet]. Wolters Kluwer Medknow Publications; 2023 [cited 2025 Mar 6];28:10. 10.4103/IJNMR.IJNMR_65_22 10.4103/ijnmr.ijnmr_65_22 PMC10215553 37250942

[4] Ferreira AF, Soares M, Almeida-Santos T, Ramalho-Santos J, Sousa AP. Aging and oocyte competence: a molecular cell perspective. WIREs mechanisms of disease [Internet]. WIREs Mech Dis; 2023 [cited 2025 Mar 6];15. 10.1002/WSBM.1613 10.1002/wsbm.1613 37248206

[5] Cimadomo D, Fabozzi G, Vaiarelli A, Ubaldi N, Ubaldi FM, Rienzi L. Impact of maternal age on oocyte and embryo competence. Front Endocrinol (Lausanne) [Internet]. Frontiers Media S.A.; 2018 [cited 2025 Mar 6];9:382238. 10.3389/FENDO.2018.00327/PDF 10.3389/fendo.2018.00327 PMC6033961 30008696

[6] Nybo Andersen AM, Wohlfahrt J, Christens P, Olsen J, Melbye M. Maternal age and fetal loss: population based register linkage study. BMJ : British Medical Journal [Internet]. 2000 [cited 2025 Mar 6];320:1708. 10.1136/BMJ.320.7251.1708 10.1136/bmj.320.7251.1708 PMC27416 10864550

[7] Devesa-Peiro A, Sebastian-Leon P, Parraga-Leo A, Pellicer A, Diaz-Gimeno P. Breaking the ageing paradigm in endometrium: endometrial gene expression related to cilia and ageing hallmarks in women over 35 years. Hum Reprod [Internet]. Hum Reprod; 2022 [cited 2025 Mar 6];37:762–76. 10.1093/HUMREP/DEAC010 10.1093/humrep/deac010 35085395

[8] Wu Y, Li M, Zhang J, Wang S. Unveiling uterine aging: much more to learn. Ageing Res Rev [Internet]. Ageing Res Rev; 2023 [cited 2025 Mar 6];86. 10.1016/J.ARR.2023.101879 10.1016/j.arr.2023.101879 36764360

[9] Pathare ADS, Loid M, Saare M, Gidlöf SB, Esteki MZ, Acharya G, et al. Endometrial receptivity in women of advanced age: an underrated factor in infertility. Hum Reprod Update [Internet]. Oxford Academic; 2023 [cited 2025 Mar 6];29:773–93. 10.1093/HUMUPD/DMAD019 10.1093/humupd/dmad019 PMC10628506 37468438

[10] Lee Y, Bohlin J, Page CM, Nustad HE, Harris JR, Magnus P, et al. Associations between epigenetic age acceleration and infertility. Human Reproduction. Oxford University Press; 2022;37:2063–74. 10.1093/HUMREP/DEAC147 10.1093/humrep/deac147 PMC9433848 35771672

[11] Deryabin PI, Borodkina A V. Epigenetic clocks provide clues to the mystery of uterine ageing. Hum Reprod Update [Internet]. Oxford Academic; 2023 [cited 2025 Mar 6];29:259–71. 10.1093/HUMUPD/DMAC042 10.1093/humupd/dmac042 36515535

[12] Blaha GC, Leavitt WW. Ovarian steroid dehydrogenase histochemistry and circulating progesterone in aged golden hamsters during the estrous cycle and pregnancy. Biol Reprod [Internet]. Oxford Academic; 1974 [cited 2025 Mar 6];11:153–61. 10.1095/BIOLREPROD11.2.153 10.1095/biolreprod11.2.153 4457131

[13] Ohta Y. Age-related decline in deciduogenic ability of the rat uterus. Biol Reprod [Internet]. Oxford Academic; 1987 [cited 2025 Mar 6];37:779–85. 10.1095/BIOLREPROD37.4.779 10.1095/biolreprod37.4.779 3480007

[14] Gupta P, Banker M, Patel P, Joshi B. A study of recipient related predictors of success in oocyte donation program. J Hum Reprod Sci [Internet]. 2012 [cited 2025 Mar 6];5:252–7. 10.4103/0974-1208.106336 10.4103/0974-1208.106336 PMC3604831 23531511

[15] Cano F, Simon C, Remohi J, Pellicer A. Effect of aging on the female reproductive system: evidence for a role of uterine senescence in the decline in female fecundity. Fertil Steril [Internet]. Elsevier Inc.; 1995 [cited 2025 May 4];64:584–9. 10.1016/S0015-0282(16)57797-8 10.1016/s0015-0282(16)57797-8 7543864

[16] Chen P, Yang M, Wang Y, Guo Y, Liu Y, Fang C, et al. Aging endometrium in young women: molecular classification of endometrial aging-based markers in women younger than 35 years with recurrent implantation failure. J Assist Reprod Genet [Internet]. Springer; 2022 [cited 2025 Mar 6];39:2143–51. 10.1007/S10815-022-02578-X/METRICS 10.1007/s10815-022-02578-x PMC9475014 35881273

[17] Garrido-Gómez T, Ruiz-Alonso M, Blesa D, Diaz-Gimeno P, Vilella F, Simón C. Profiling the gene signature of endometrial receptivity: clinical results. Fertil Steril. Elsevier; 2013;99:1078–85. 10.1016/J.FERTNSTERT.2012.12.005 10.1016/j.fertnstert.2012.12.005 23312228

[18] Talbi S, Hamilton AE, Vo KC, Tulac S, Overgaard MT, Dosiou C, et al. Molecular phenotyping of human endometrium distinguishes menstrual cycle phases and underlying biological processes in normo-ovulatory women. Endocrinology [Internet]. Endocrinology; 2006 [cited 2025 Mar 6];147:1097–121. 10.1210/EN.2005-1076 10.1210/en.2005-1076 16306079

[19] Altmäe S, Koel M, Võsa U, Adler P, Suhorutšenko M, Laisk-Podar T, et al. Meta-signature of human endometrial receptivity: a meta-analysis and validation study of transcriptomic biomarkers. Sci Rep [Internet]. Nature Publishing Group; 2017 [cited 2025 May 4];7. 10.1038/S41598-017-10098-3 10.1038/s41598-017-10098-3 PMC5577343 28855728

[20] Díaz-Gimeno P, Horcajadas JA, Martínez-Conejero JA, Esteban FJ, Alamá P, Pellicer A, et al. A genomic diagnostic tool for human endometrial receptivity based on the transcriptomic signature. Fertil Steril [Internet]. Elsevier Inc.; 2011 [cited 2025 May 4];95. 10.1016/j.fertnstert.2010.04.063 10.1016/j.fertnstert.2010.04.063 20619403

[21] Wilcox AJ, Baird DD, Weinberg CR. Time of implantation of the conceptus and loss of pregnancy. N Engl J Med [Internet]. N Engl J Med; 1999 [cited 2025 May 4];340:1796–9. 10.1056/NEJM199906103402304 10.1056/NEJM199906103402304 10362823

[22] Valdes CT, Schutt A, Simon C. Implantation failure of endometrial origin: it is not pathology, but our failure to synchronize the developing embryo with a receptive endometrium. Fertil Steril [Internet]. Elsevier Inc.; 2017 [cited 2025 May 4];108:15–8. 10.1016/j.fertnstert.2017.05.033 10.1016/j.fertnstert.2017.05.033 28668151

[23] Díaz-Gimeno P, Horcajadas JA, Martínez-Conejero JA, Esteban FJ, Alamá P, Pellicer A, et al. A genomic diagnostic tool for human endometrial receptivity based on the transcriptomic signature. Fertil Steril. Elsevier Inc.; 2011;95. 10.1016/j.fertnstert.2010.04.063 10.1016/j.fertnstert.2010.04.063 20619403

[24] Ruiz-Alonso M, Blesa D, Díaz-Gimeno P, Gómez E, Fernández-Sánchez M, Carranza F, et al. The endometrial receptivity array for diagnosis and personalized embryo transfer as a treatment for patients with repeated implantation failure. Fertil Steril. 2013;100:818–24. 10.1016/j.fertnstert.2013.05.004 10.1016/j.fertnstert.2013.05.004 23756099

[25] Simon C, Gomez C, Cabanillas S, Vladimirov IK, Castillon G, Giles J, et al. In vitro fertilization with personalized blastocyst transfer versus frozen or fresh blastocyst transfer: a multicenter, randomized clinical trial. Fertil Steril. Elsevier BV; 2019;112:e56–7. 10.1016/J.FERTNSTERT.2019.07.273

[26] Doyle N, Jahandideh S, Hill MJ, Widra EA, Levy M, Devine K. Effect of timing by endometrial receptivity testing vs standard timing of frozen embryo transfer on live birth in patients undergoing in vitro fertilization: a randomized clinical trial. JAMA [Internet]. JAMA; 2022 [cited 2025 Mar 6];328:2117–25. 10.1001/JAMA.2022.20438 10.1001/jama.2022.20438 PMC9856480 36472596

[27] Bassil R, Casper R, Samara N, Hsieh TB, Barzilay E, Orvieto R, et al. Does the endometrial receptivity array really provide personalized embryo transfer? J Assist Reprod Genet. Springer New York LLC; 2018;35:1301–5. 10.1007/s10815-018-1190-9 10.1007/s10815-018-1190-9 PMC6063827 29737471

[28] Huang D, Flynn E, Almonte-Loya A, Davidson B, Chan M, Casillas A, et al. A positive ReceptivaDx result for BCL6 does not correlate with abnormal ERA results or decreased expression of receptivity-associated markers: two sides of the endometrial receptivity coin in fertility evaluation and treatment. F and S Science [Internet]. Elsevier Inc.; 2025 [cited 2025 Oct 9];6:55–64. 10.1016/j.xfss.2024.10.005 10.1016/j.xfss.2024.10.005 PMC11829826 39419175

[29] Bosch A, Hipp HS. No endometrial receptivity assay of enlightenment for recurrent implantation failure. Fertil Steril [Internet]. Fertil Steril; 2023 [cited 2024 Oct 13];119:239–40. 10.1016/J.FERTNSTERT.2022.12.007 10.1016/j.fertnstert.2022.12.007 36496083

[30] Mei Y, Wang Y, Ke X, Liang X, Lin Y, Wang F. Does endometrial receptivity array improve reproductive outcomes in euploid embryo transfer cycles? A systematic review. Front Endocrinol (Lausanne) [Internet]. Frontiers Media SA; 2023 [cited 2024 Oct 14];14:1251699. 10.3389/fendo.2023.1251699 10.3389/fendo.2023.1251699 PMC10641275 37964969

[31] Riestenberg C, Kroener L, Quinn M, Ching K, Ambartsumyan G. Routine endometrial receptivity array in first embryo transfer cycles does not improve live birth rate. Fertil Steril [Internet]. Fertil Steril; 2021 [cited 2024 Oct 13];115:1001–6. 10.1016/J.FERTNSTERT.2020.09.140 10.1016/j.fertnstert.2020.09.140 33461752

[32] Neves AR, Devesa M, Martínez F, Garcia-Martinez S, Rodriguez I, Polyzos NP, et al. What is the clinical impact of the endometrial receptivity array in PGT-A and oocyte donation cycles? J Assist Reprod Genet [Internet]. Springer New York LLC; 2019 [cited 2025 Sep 3];36:1901–8. 10.1007/S10815-019-01535-5 10.1007/s10815-019-01535-5 PMC6730969 31352621

[33] Loid M, Obukhova D, Kask K, Apostolov A, Meltsov A, Tserpelis D, et al. Aging promotes accumulation of senescent and multiciliated cells in human endometrial epithelium. Hum Reprod Open [Internet]. Oxford University Press; 2024 [cited 2025 Sep 6];2024. 10.1093/HROPEN/HOAE048 10.1093/hropen/hoae048 PMC11344589 39185250

[34] Yeh JS, Steward RG, Dude AM, Shah AA, Goldfarb JM, Muasher SJ. Pregnancy outcomes decline in recipients over age 44: an analysis of 27,959 fresh donor oocyte in vitro fertilization cycles from the Society for Assisted Reproductive Technology. Fertil Steril [Internet]. Fertil Steril; 2014 [cited 2025 Mar 13];101. 10.1016/J.FERTNSTERT.2014.01.056 10.1016/j.fertnstert.2014.01.056 24626061

[35] Vitagliano A, Paffoni A, Viganò P. Does maternal age affect assisted reproduction technology success rates after euploid embryo transfer? A systematic review and meta-analysis. Fertil Steril [Internet]. Elsevier Inc.; 2023 [cited 2025 Sep 6];120:251–65. 10.1016/J.FERTNSTERT.2023.02.036 10.1016/j.fertnstert.2023.02.036 36878347

[36] Pathare ADS, Loid M, Saare M, Gidlöf SB, Esteki MZ, Acharya G, et al. Endometrial receptivity in women of advanced age: an underrated factor in infertility. Hum Reprod Update [Internet]. Hum Reprod Update; 2023 [cited 2025 Feb 26];29:773–93. 10.1093/HUMUPD/DMAD019 10.1093/humupd/dmad019 PMC10628506 37468438

[37] Sher G, Herbert C, Maassarani G, Jacobs MH. Assessment of the late proliferative phase endometrium by ultrasonography in patients undergoing in-vitro fertilization and embryo transfer (IVF/ET). Human Reproduction [Internet]. Oxford University Press; 1991 [cited 2025 May 4];6:232–7. 10.1093/OXFORDJOURNALS.HUMREP.A137312 10.1093/oxfordjournals.humrep.a137312 2056019

[38] Randolph JF, Sowers M, Bondarenko I V., Harlow SD, Luborsky JL, Little RJ. Change in estradiol and follicle-stimulating hormone across the early menopausal transition: effects of ethnicity and age. Journal of Clinical Endocrinology and Metabolism [Internet]. J Clin Endocrinol Metab; 2004 [cited 2025 May 4];89:1555–61. 10.1210/JC.2003-031183 10.1210/jc.2003-031183 15070912

[39] Burger HG, Dudley EC, Hopper JL, Groome N, Guthrie JR, Green A, et al. Prospectively measured levels of serum follicle-stimulating hormone, estradiol, and the dimeric inhibins during the menopausal transition in a population-based cohort of women. J Clin Endocrinol Metab [Internet]. J Clin Endocrinol Metab; 1999 [cited 2025 May 4];84:4025–30. 10.1210/JCEM.84.11.6158 10.1210/jcem.84.11.6158 10566644

[40] Soares SR, Troncoso C, Bosch E, Serra V, Simón C, Remohí J, et al. Age and uterine receptiveness: predicting the outcome of oocyte donation cycles. J Clin Endocrinol Metab [Internet]. J Clin Endocrinol Metab; 2005 [cited 2025 Mar 13];90:4399–404. 10.1210/JC.2004-2252 10.1210/jc.2004-2252 15797956

[41] Toner JP, Grainger DA, Frazier LM. Clinical outcomes among recipients of donated eggs: an analysis of the U.S. national experience, 1996-1998. Fertil Steril [Internet]. Fertil Steril; 2002 [cited 2025 Mar 13];78:1038–45. 10.1016/S0015-0282(02)03371-X 10.1016/s0015-0282(02)03371-x 12413990

[42] Doyle N, Jahandideh S, Hill MJ, Widra EA, Levy M, Devine K. Effect of timing by endometrial receptivity testing vs standard timing of frozen embryo transfer on live birth in patients undergoing in vitro fertilization: a randomized clinical trial. JAMA [Internet]. American Medical Association; 2022 [cited 2025 Sep 3];328:2117–25. 10.1001/JAMA.2022.20438 10.1001/jama.2022.20438 PMC9856480 36472596

[43] Zolfaroli I, Monzó Miralles A, Hidalgo-Mora JJ, Marcos Puig B, Rubio Rubio JM. Impact of endometrial receptivity analysis on pregnancy outcomes in patients undergoing embryo transfer: a systematic review and meta-analysis. J Assist Reprod Genet [Internet]. Springer; 2023 [cited 2025 Sep 4];40:985–94. 10.1007/S10815-023-02791-2 10.1007/s10815-023-02791-2 PMC10239419 37043134

[44] Eisman LE, Pisarska MD, Wertheimer S, Chan JL, Akopians AL, Surrey MW, et al. Clinical utility of the endometrial receptivity analysis in women with prior failed transfers. J Assist Reprod Genet [Internet]. J Assist Reprod Genet; 2021 [cited 2025 Sep 28];38:645–50. 10.1007/S10815-020-02041-9 10.1007/s10815-020-02041-9 PMC7910393 33454901

[45] Chae-Kim J, Doyle N, Jahandideh S, O’Brien JE, Widra EA, Levy M, et al. Evaluating the endometrial receptivity assay: a nested diagnostic accuracy study within the synchrony randomized clinical trial. Fertil Steril [Internet]. Elsevier Inc.; 2023 [cited 2025 Sep 28];120:1255–6. 10.1016/j.fertnstert.2023.09.004 10.1016/j.fertnstert.2023.09.004 37714255

[46] Barrenetxea G, Romero I, Celis R, Abio A, Bilbao M, Barrenetxea J. Correlation between plasmatic progesterone, endometrial receptivity genetic assay and implantation rates in frozen-thawed transferred euploid embryos. A multivariate analysis. European Journal of Obstetrics and Gynecology and Reproductive Biology [Internet]. Elsevier Ireland Ltd; 2021 [cited 2025 Sep 3];263:192–7. 10.1016/J.EJOGRB.2021.05.047 10.1016/j.ejogrb.2021.05.047 34229182

[47] Devine K, Richter KS, Jahandideh S, Widra EA, McKeeby JL. Intramuscular progesterone optimizes live birth from programmed frozen embryo transfer: a randomized clinical trial. Fertil Steril [Internet]. Elsevier Inc.; 2021 [cited 2025 Sep 4];116:633–43. 10.1016/j.fertnstert.2021.04.013 10.1016/j.fertnstert.2021.04.013 33992421

[48] Miles RA, Paulson RJ, Lobo RA, Press MF, Dahmoush L, Sauer M V. Pharmacokinetics and endometrial tissue levels of progesterone after administration by intramuscular and vaginal routes: a comparative study. Fertil Steril [Internet]. Elsevier Inc.; 1994 [cited 2025 Oct 9];62:485–90. 10.1016/s0015-0282(16)56935-0 10.1016/s0015-0282(16)56935-0 8062942

[49] Doyle N, Jahandideh S, Hill MJ, Widra EA, Levy M, Devine K. Effect of timing by endometrial receptivity testing vs standard timing of frozen embryo transfer on live birth in patients undergoing in vitro fertilization: a randomized clinical trial. JAMA [Internet]. American Medical Association; 2022 [cited 2025 May 4];328:2117–25. 10.1001/JAMA.2022.20438 10.1001/jama.2022.20438 PMC9856480 36472596

