# Supplementary material for: Endometrial aging and uterine receptivity: endometrial receptivity analysis (ERA) outcomes in female patients of diverse age groups
Source: J Assist Reprod Genet. 2026 Feb 9;43(4):1169–77. doi: 10.1007/s10815-026-03824-2 (PMC13103149; doi:10.1007/s10815-026-03824-2)
Supplement: Supplementary file 1 — Supplementary file1 (DOCX 16.9 KB) [file 10815_2026_3824_MOESM1_ESM.docx]

| **Supplemental Table 1. Outcomes of Subsequent Transfer after EMB (N=192)** | | | | | |
| --- | --- | --- | --- | --- | --- |
|  | **<35 (N=35)** | **35-37 (N=58)** | **38-40 (N=53)** | **>41 (N=59)** | **p-value** |
| **Implantation Rate** | 25/35 (71) | 29/51 (57) | 35/51 (69) | 30/55 (55) | 0.249 |
| **Clinical Pregnancy Rate** | 21/35 (60) | 26/51 (51) | 30/51 (59) | 24/55 (44) | 0.338 |
| **Live Birth Rate** | 18/35 (51) | 24/51 (47) | 25/51 (49) | 23/55 (42) | 0.814 |
| Data reported as n/N (%) | | | | | |

| **Supplemental Table 2. Outcomes of Subsequent Transfer after EMB if ERA was Non-Receptive (N=38)** | | | | | |
| --- | --- | --- | --- | --- | --- |
|  | **<35 (N=7)** | **35-37 (N=10)** | **38-40 (N=9)** | **>41 (N=12)** | **p-value** |
| **Implantation Rate** | 6/7 (86) | 5/10 (50) | 6/9 (67) | 7/12 (58) | 0.498 |
| **Clinical Pregnancy Rate** | 4/7 (57) | 5/10 (50) | 5/9 (56) | 4/12 (33) | 0.695 |
| **Live Birth Rate** | 4/7 (57) | 4/10 (40) | 5/9 (56) | 3/12 (25) | 0.440 |
| Data reported as n/N (%) | | | | | |

| **Supplemental Table 3. Proportion of Non-Receptive ERA by Age - Programmed Only (N=170)** | | | | | |
| --- | --- | --- | --- | --- | --- |
|  | **<35 (N=25)** | **35-37 (N=45)** | **38-40 (N=45)** | **>41 (N=55)** | **p-Value** |
| **Non-Receptive ERA** | 2 (8.0%) | 7 (15.6%) | 7 (15.6%) | 13 (23.6%) | 0.25 |
| Pre-Receptive | 2 (8.0%) | 6 (13.3%) | 5 (11.1%) | 13 (23.6%) |  |
| Post-Receptive | 0 (0%) | 1 (2.2%) | 2 (4.4%) | 0 (0%) |  |
| **Receptive ERA** | 23 (92.0%) | 38 (84.4%) | 38 (84.4%) | 42 (76.4%) |  |
